# The quest for molecular markers indicating root growth in microbially treated tomato (*Solanum lycopersicum*) plants

**DOI:** 10.1093/femsec/fiaf063

**Published:** 2025-06-18

**Authors:** Leonard S van Overbeek, Stefan Aanstoot, Erik Esveld, Lina Russ, Beatriz Andreo Jimenez

**Affiliations:** Wageningen University and Research (WUR), Radix building (building 107), Droevendaalsesteeg 1, 6700AB Wageningen, The Netherlands; Wageningen University and Research (WUR), Radix building (building 107), Droevendaalsesteeg 1, 6700AB Wageningen, The Netherlands; Wageningen University and Research (WUR), Radix building (building 107), Droevendaalsesteeg 1, 6700AB Wageningen, The Netherlands; Wageningen University and Research (WUR), Radix building (building 107), Droevendaalsesteeg 1, 6700AB Wageningen, The Netherlands; Wageningen University and Research (WUR), Radix building (building 107), Droevendaalsesteeg 1, 6700AB Wageningen, The Netherlands

**Keywords:** microbial inoculant, RNAseq, root development, root growth indicator, stone wool, tomato plants, *Verrucomicrobium*, X-ray microtomography

## Abstract

Roots are essential plant organs for anchorage in soil, uptake of water with nutrients, storage of photosynthates, and microbial interactions. More knowledge on microorganisms stimulating root growth is needed to control root development of cultured plants. A marker-assisted approach would facilitate vast screenings of microbes for eventual effects on root development. It was aimed to select for transcripts that report on root growth stimulation at the early tomato plant growth stage upon microbial treatments. Microbially treated tomato (*Solanum lycopersicum*) plants were cultivated in stone wool slabs and screened for genes that increased or decreased in differential expression upon increased root growth, by RNAseq. Expression of 21 selected genes was measured by quantitative polymerase chain reaction (qPCR) in relation with stimulated root growth, recorded by X-ray microtomography, of microbially treated tomato plants cultivated in stone wool blocks. Two genes were identified of which expression significantly correlated with high measured root length, and for one also with high measured shoot wet and dry weight. The translated products, both involved in modulation of Rubisco activity, were a chloroplast-localized acetyltransferase (SlSNAT2) and a Rubisco activase (Rca). Transcripts whose translated products modulate Rubisco activity can serve as candidates for reporting on early root development upon microbial inoculation.

## Teaser

A molecular marker-assisted approach was designed for fast screening among microbial inoculants that stimulate root growth in tomato plants.

## Introduction

Roots are essential plant organs for anchorage in soil, uptake of water with dissolved nutrients, storage of photosynthates, and for interactions with soil microorganisms (Atkinson et al. 2014, Hoang et al. 2020). These functions all are important to maintain a productive and healthy status of arable plants. Plants benefit from comprehensive root surfaces by intense interactions with beneficial microorganisms present in soils (Balestrini et al. 2024). Extensive root growth will overall result in better plant development and increased resilience to different forms of stress (Hoang et al. 2020). Many environmental factors play a role in root growth and development and there is still no complete overview on all factors contributing to root growth (Hardtke 2006, Osmont et al. 2007).

Plant-associated microbes support plant development by root growth stimulation under suboptimal circumstances, such as in parched soils, or in soils limited in P or N (Osmont et al. 2007, Estrada-Bonilla et al. 2012, Eltlbany et al. 2019). Certain microbial strains are applied as active ingredients in products meant to stimulate root growth in arable plants (Ortíz-Castro et al. 2009, Verbon and Liberman 2016). In specific, root growth stimulants can be relevant for plants cultivated in alternative growth matrices, such as stone wool, that are virtually void of indigenous microbes (Mendes et al. 2013, Bakker et al. 2018). High value food and ornamental plants are routinely cultivated in stone wool blocks in greenhouses in the Netherlands and microbial inoculants could contribute to root growth and development. Root growth stimulants act as biostimulants because they can increase nutrient uptake and tolerance to abiotic stresses. Marketing of biostimulants within the EU is harmonized and should comply with EU regulation EU 2019/1009 on fertilizing products. In the current situation, only four microbial groups can be registered as biostimulants, i.e. *Azospirillum, Azotobacter*, and *Rhizobium* spp and mycorrhizal fungi. More information on the role of other microbial species in root development is therefore needed to offer new perspectives to growers for root growth control in their crop production practices.

To investigate the impact of microbial inoculants on root growth, it is important to accurately measure root systems (Atkinson et al. 2018). For root growth studies done in soils, it is difficult to keep root systems intact and not to cause any disturbances in root structures (Poole 2017). Measurements causing disturbances in root structures provide incomplete information on root capacity for nutrient uptake and microbial interactions. In the laboratory, root structures can be measured over time, but then plants must be grown under artificial circumstances (Atkinson et al. 2014). New analytical tools would therefore be required to investigate root growth in its matrix under realistic growth conditions, preferably nondestructive, to allow multiple root measurements per plant growth cycle (Flavel et al. 2012, Tracy et al. 2012). These types of measurements could offer better insight in the adaptation of plants by modification of their root systems. X-ray microtomography is such a novel approach (Tracy et al. 2012, Poole 2017), although still confronted with technical constrains in visualization of root systems in natural soils (Zappala et al. 2013). However, it is possible to measure entire root systems and to quantify multiple traits in root architecture that are important for plant growth (Mooney et al. 2011, Flavel et al. 2012, Tracy et al. 2012). Total root system analysis can therefore be used as a tool to study the impact of microorganisms, derived from plant microbiomes, on stimulation of root growth (Verbon and Liberman 2016, Poole 2017).

The modes of action of many microorganisms isolated as root growth stimulants from soil and plant microbiomes are unknown (Bulgarelli et al. 2012, Mendes et al. 2013, Yu and Hochholdinger 2018). For commercial purposes, the search for individual strains or mixtures thereof (SynComs) in soil and plant microbiomes would be relevant for development of new products that can be applied as root growth stimulants in sustainable practices. Although novel insight on root architectural changes can be offered by X-ray microtomography, this method cannot be applied in high throughput screenings for root growth stimulation in plants treated with microbial agents. As alternative, molecular markers in plants, that are indicative for stimulated root growth, can be an option to screen among vast numbers of microbially treated plants for identification of root growth-stimulating microbial strains. Transcription-based markers could monitor early stages in root growth, whereas their translated products do not necessarily need to be involved in root architectural processes (Verbon and Liberman 2016, Hoang et al. 2020). For the quest to such transcripts in plants, an untargeted approach, such as RNAseq, would be an adequate tool.

The objective of this study was to search for transcriptional factors that report on root growth upon microbial treatments at the early plant growth stage. For that purpose, we used a set of plant-associated bacterial strains that originated from surfaces and internal compartments of different plant species. By individual applications of different microbial strains (bacteria and a fungus in a commercial product), we attempted to stimulate root growth in our model plant tomato (*Solanum lycopersicum*), an important food crop in Dutch greenhouse production, via different regulatory networks (Ortíz-Castro et al. 2009, Verbon and Liberman 2016). The relationship between root growth stimulation and differential expression of genes was investigated using the combination of *in vitro* root growth measurements in stone wool slabs and RNAseq analysis on stem base samples. In a subsequent step, a selected set of primer systems, targeting transcripts of genes that are strongly increased or reduced in expression upon root growth stimulation, was evaluated on microbially treated plants grown in stone wool blocks of which the total root sizes were recorded by X-ray microtomography.

## Materials and methods

### Experimental design

The experiment consisted of two phases, a first phase wherein genes were selected that were differentially expressed upon stimulated root growth, and a second wherein a selected set of transcripts was evaluated for correlation with root and shoot growth of microbially treated tomato plants (Fig. S1). In phase 1, tomato plants were individually grown in stone wool slabs and a total of 16 microbial and control treatments were applied on five replicate plants per treatment in two independent experiments. RNAseq analysis was performed on pooled RNA samples of each treatment, separate for the replicate experiments. In phase 2, tomato plants were individually grown in stone wool blocks and a total of five microbial and control treatments were applied on five replicate plants per treatment in one single experiment. Total root size was recorded by X-ray microtomography and gene expression of 21 selected transcripts was quantified by qPCR on plant RNA extracts.

### Microbial strains

Microbial strains, differing in taxonomical identity and originating from different sources, were used in this study (Table 1). Two strains were used as references because these have already been applied as active compounds in products. *Trichoderma afroharzianum* strain T-22 was used in a formulated form, Trianum-P (Koppert Biological Systems BV, Netherlands), and *Bacillus velezensis* strain FZB42 in a nonformulated form and this strain is the active compound of RhizoVital (Abiteb, Germany). Further, three strains (E353, E390, and E394), characterized as endophytes and originating from surface-sterilized stems of greenhouse-cultivated tomato plants cv. Moneymaker, were used. All other bacterial strains originated from roots and rhizosphere soils of different plant species. Among the group of bacteria, strain identities affiliated with different taxonomical phyla including *Actinobacteria* (E394), *Bacteroidetes* (E353), *Firmicutes* (FZB42), *Proteobacteria* (*Betaproteobacteria*, PsJN, E39, and *Gammaproteobacteria*, D5/23, R034, R035, R043, R082, and R175), and *Verrucomicrobium* subdivision 1 (CHC8, C20, and Z35).

**Table 1.** Microbial strains used for tomato plant treatments.

Strain	Taxonomic identity	Origin/reference
T-22	*T. afroharzianum*	Trianum-P, Koppert biological systems, Netherlands
FZB42	*B. velezensis*	Plant pathogen infested soil (Krebs et al. 1998), active component of Rhizovital Abiteb, Germany
PsJN	*Paraburkholderia phytofirmans*	Onion roots (Sessitsch et al. 2005)
D5/23	*Kosakonia radicincitans*	Winter wheat (Kämpfer et al. 2005)
R034	*Klebsiella variicola*	Rice roots (Hardoim et al. 2011)
R035	*Enterobacter* sp.	Rice roots (Hardoim et al. 2011)
R043	*Citrobacter freundii*	Rice roots (Hardoim et al. 2011)
R082	*Enterobacter* sp.	Rice roots (Hardoim et al. 2011)
R175	*Pseudomonas* sp.	Rice roots (Hardoim et al. 2011)
E353	*Mucilaginibacter pocheonensis*	Surface-sterilized tomato stem, unpublished
E390	*Acidovorax avenae*	Surface-sterilized tomato stem, unpublished
E394	*Kokuria kristinae*	Surface-sterilized tomato stem, unpublished
CHC8	*Verrucomicrobium* subdivision 1, *Candidatus* genus *Rhizospheria*	Leek rhizosphere (Nunes da Rocha et al. 2010)
C20	*Verrucomicrobium* subdivision 1, *Luteolibacter*	Potato rhizosphere (Nunes da Rocha et al. 2010)
Z35	*Verrucomicrobium* subdivision 1, *Candidatus* genus *Rhizospheria*	Potato rhizosphere (Nunes da Rocha et al. 2010)

*Verrucomicrobium* subdivision 1 strains were cultivated on R2A (Sigma Aldrich) for 7–10 days at 25°C, because of their recalcitrance to grow in liquid broth (Nunes da Rocha et al. 2011). All other bacterial strains were overnight cultivated in one-tenth strength tryptic soy broth (ThermoFisher Scientific, Waltham, Ma) at 25°C under rotation. Cells from plates were harvested, by scraping from the agar surface with a sterilized spatula, in quarter strength Ringer solution (¼ Ringer solution; Oxoid, UK) and cell suspensions of all bacterial strains were centrifuged for 5 min at 10 000 × *g*. Resulting cell pellets were three times washed in ¼ Ringer solution by repeated resuspension and centrifugation steps and finally diluted in tomato nutrient solution (TNS; Voogt and Sonneveld 1997). Spores of *T. afroharzianum* T-22 (Trianum-P) were diluted in TNS as recommended by the manufacturer, and all bacterial cell and strain T-22 spore suspensions were set at 10^8^ CFU ml^−1^ using TNS.

### Tomato plant treatments and growth

Tomato plants (cultivar Delioso F1, Rijk Zwaan, Netherlands) were grown in stone wool (Grodan Rockwool BV, NL) slabs of 12 (W) × 12 (H) × 1 (D) cm, in phase 1, or in blocks of 10 (W) × 11.2 (H) × 20 (D) cm in size, in phase 2. Slabs were covered with two transparent plastic sheets on both large sides for recording root growth over time, and blocks were wrapped in white plastic foil on four sides, to avoid excessive water evaporation. Slabs and blocks were soaked in TNS until complete saturation. Then, seeds were placed on top of open sides of slabs and blocks and individually treated with 100 µl bacterial cell or strain T-22 spore suspension (Trianum-P treatment). Control plants were treated with TNS only. All microbial and control treatments were repeated on emerging seeds after 3 days. Seven slabs of the same microbial or control treatment were placed in one tray and only the inner five slabs were used for recording root development over time and RNA extraction. Blocks were individually placed on supporting trays and TNS was administered to slabs and blocks from below, every 3 days. Seeds in slabs were allowed to germinate and to grow out to plants in a plant growth cabinet for 21 d (Weisstechnik, USA) and in blocks in the greenhouse for 17 d, all under the same day/night regime of 16 h light at 25°C/8 h dark at 20°C, at a constant air humidity of 70%.

### Root and shoot measurements in stone wool slabs and blocks

Root sizes and number of side branches were recorded in stone wool slabs at different time periods after seeding, namely at days 7, 10, 14, and 21. Therefore, roots visible under the transparent sheets on both sides of the slabs were marked with different colours for each time period. Then, sheets were scanned and the cumulative curved length was determined using optimized colour separation and image skeletonization procedures created in Matlab (MathWorks Inc, MA). Plants were destructively sampled at day 21 by cutting shortly above the stone wool surface. The transition zone between roots and stem at the first 3 cm above the stone wool surface, and further denoted as ‘stem base’, of all plants were sampled and snap-frozen for 10 s in liquid nitrogen and further stored at −70°C for later RNA extraction. Further, length, fresh weight, and number of compound leaves of the shoots were measured.

The total length of root systems in stone wool blocks was determined via X-ray microtomography at 17 d after sowing. Therefore, plants were destructively sampled by cutting off shoots as described before. Three centimetre stem base samples were taken from the shoots, snap-frozen and stored at −70°C for later RNA extraction. Fresh weight of the separated shoots were immediately determined, after which samples were overnight dried at 60°C for dry weight measurement. The blocks still containing intact root systems were dehydrated for 40 min via capillary drainage on a presoaked stacked column of stone wool blocks of 1.5 m in height and thus dehydrated blocks were stored overnight at 50% RH. Then, individual stone wool blocks with roots were scanned at 70 µm resolution (Phoenix v|tome|x m, General Electric, Germany) during a time frame of 45 min. Reconstructed 3D images of all root systems in stone wool blocks were identically processed using Avizo V9.2 (Thermo Fisher Scientific, MA) to visualize roots via a combination of thresholding and morphological filtering. Remaining capillary water clusters could be eliminated from the segmented root system by skeletonization and deletion of small and thin segments.

### Recovery of introduced microbial strains from roots in stone wool blocks

After X-ray microtomography, roots were separated from three randomly selected stone wool blocks for quantitative detection, by qPCR, of strains Z35, FZB42, and R043, and for recovery by cultivation, of strains T-22, FZB42, and R043. Therefore, roots from each block were split into two batches for DNA extraction and microbial recovery.

DNA was extracted from 100 mg frozen (−20°C) root samples using the DNeasy PowerSoil Kit (Qiagen, Germany), following the instruction provided by the manufacturer. DNA was quantified using the Qubit quantitation platform (Thermo Fisher Scientific) and 10 fg of root DNA was used as target in quantitative polymerase chain reactions (qPCRs) using Takara TB Green Premix Ex Taq II kit (Takara Bio Inc., Japan), combined with the primers described in Table S1. For calibration, synthetic double-stranded DNA targets (gBlocks), complementary to each primer system, were used. Reactions were run in an Applied Biosystems 7500 real-time PCR system (Thermo Fisher Scientific), using a thermal cycling regime of one cycle at 95°C for 2 min, followed by 40 cycles at 95°C for 10 s and 60°C for 45 s. During the program, fluorescence was recorded, and cycle threshold (Ct) values established. In addition, dissociation curves of the amplicons were made at the end of the program at a temperature range between 60°C and 95°C.

For recovery of strain T-22 from Trianum-P-treated plants, root parts of between one and three cm in length were placed onto potato dextrose agar (PDA; ThermoFisher Scientific) in Petridishes. For recovery of strains FZB42 and R043 from treated plants, samples composed of 5 g root parts were transferred to BioReba extraction bags (BioReba AG, Switzerland) containing 5 ml of ¼ Ringer solution, after which roots were macerated and suspensions plated onto R2A. All plates were incubated at 25°C for 4 days, after which they were inspected for presence of colonies with morphologies identical to the introduced strains.

### RNA extraction from stem base samples, reverse transcription, sequencing, and data processing

Total RNA was extracted from all snap-frozen and stored stem base samples of plants grown in stone wool slabs and blocks. Therefore, samples were homogenized by bead beating for 30 s at 8000 RPM using a Precellys® bead beater (Bertin Technologies, France). Obtained homogenates were kept on ice during all RNA extraction steps, using the RNeasy plant mini kit (Qiagen). DNA was removed from all RNA samples using RNAse-free DNAse (DNAse I, amplification grade, Thermo Fisher Scientific). RNA samples, free of DNA, were stored at −70°C for later RNAseq (from plants grown in slabs) and qPCR (from plants grown in blocks) analyses.

For RNAseq analysis, a total of 32 RNA extracts (16 pooled RNA samples of five plants per treatment, in replicate from the two separate experiments) were used. Plant mRNA samples were enriched by polyA mRNA selection using the TruSeq stranded RNA sample preparation kit (Illumina Inc, CA). Samples were fragmented and reverse transcribed into cDNA. Adapters were then ligated to final cDNA products and the obtained cDNA library was amplified, according to the protocol provided by the manufacturer. The final library, eluted to 30 µl in elution buffer, was quality checked and quantified using a Bioanalyzer 2100 DNA1000 chip (Agilent Technologies, Germany). Prepared libraries were pooled and diluted to 6 pM for TruSeq paired-end v4 DNA clustering on a one single flow cell lane using a cBot device (Illumina). Final sequencing was done on a HiSeq 2500 platform (Illumina). All steps for clustering and subsequent sequencing were carried out according to the manufacturer protocol. Reads were split per corresponding sample using CASAVA 1.8 software (Illumina), length (>30 bp) and quality trimmed (0.05, 2 ambiguous nucleotides) with CLC Genomics Workbench 11.0.1 (Qiagen) and mapped to the tomato reference genome [International tomato genome project (https://solgenomics.nethttps://solgenomics.net) version SL3.0 and ITAG3.10] using the hisat2 software excluding discordant alignments (Kim et al. 2015). Raw sequence reads from all samples were deposited in the NCBI sequence read archive under Bioproject number PRJNA644532 (https://www.ncbi.nlm.nih.gov/bioproject/PRJNA644532).

Quantitative polymerase chain reactions were conducted on reverse transcribed transcripts of 21 selected genes using RNA extracts from individual plants grown in stone wool blocks. Therefore, primers targeting transcripts of these 21 genes, further referred to as primer sets 1 through 21 (Table S2), were applied in separate qPCRs and calibrated with gBlocks, specific for each primer set. Reactions were all carried out on 75 ng template RNA in buffer solution of the Takara One-Step TB Green PrimeScript RT-PCR kit II, according to the manufacturer protocol. Reactions were run in a 7500 real time thermocycler, using the same thermocycle regime for all 21 primer sets, i.e. 5 min reverse transcription step at 42°C, followed by one denaturing step for 10 s at 95°C and 40 cycles at 95°C for 5 s and at 55°C for 45 s. Dissociation curves of all amplicons were made as described before.

### Statistical analyses

The effect of 16 microbial and control treatments on shoot (length, fresh weight, and number of compound leaves) and root (length and number of adventitious roots) parameters of tomato plants grown in stone wool slabs (phase 1) and of five microbial and control treatments on shoot (fresh and dry weight) and root (length) parameters on tomato plants grown in blocks (phase 2) were analysed by one-way ANOVA (Genstat 22nd edition, VSN international, UK), including two block treatments for the separate experiments conducted in phase 1. Differences were considered to be significant at levels of *P* ≤ .05. In case of significance, least significant difference values were calculated for making comparisons between average values. In addition, Students *t*-test was used to calculate differences in average Ct values between control and individual microbial treatments and between root length and shoot dry (in blocks only) and fresh weight values of tomato plants grown in slabs (21 d) and in blocks (17 d). Correlations between corresponding values of different shoot and root parameters from the different samples were calculated by linear regression, separate for tomato plants grown in slabs and in blocks.

Gene expression counts, obtained with htseq-count version 0.11.1 (Anders et al. 2015), were analysed with DESeq2 version 1.14.1 (Love et al. 2014) in R, comparing the effect of all individual microbial treatments with that of the control. Correlations between expression profiles of individual samples were calculated by principal component analysis (PCA) in VEGAN R package. Correlations between expression profiles and corresponding values of shoot and root parameters were calculated by canonical correspondence analysis (CCA).

Ct values from 25 reverse-transcribed RNA samples were individually analysed for each the 21 primer sets. Nondetectable values were replaced by a Ct value of 40, to a maximum of three nondetectable values per primer set, to enable statistical analyses on the data set. Datasets from four primer sets, where the number of nondetectable Ct values was higher than three, were not further used in statistical analyses. The effect of microbial and control treatments on Ct values of thus remaining 17 primer sets was determined by one-way ANOVA and comparisons between average Ct values from individual microbial treatments with that of the control was made by Students *t*-test. Correlations among Ct values from individual primers sets and of individual primer sets with corresponding shoot fresh and dry weight and root length values were calculated by linear regression.

## Results

### Effects of microbial treatments on tomato root and shoot development in stone wool slabs

**Figure 1.**
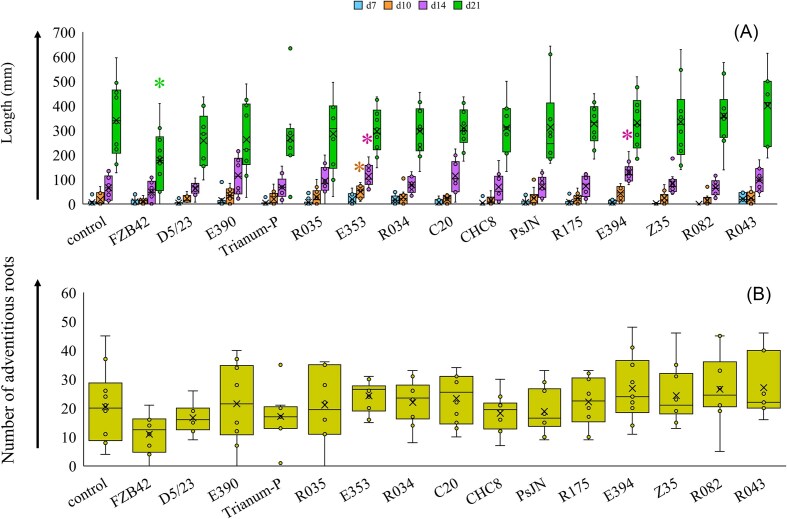
Box plot (maximum, 3rd, 2nd, and 1st quartiles, minimum values) of root length measured over time in stone wool slabs (A), and number of adventitious roots after 21 d (B). Roots of tomato plants grown in stone wool slabs (*n* = 10) were measured at 7, 10, 14, and 21 d after sowing. Box plot with * indicate significant different average value from control, as determined by Students *t*-test.

High variation in root and shoot parameters resulted from the different microbial treatments on tomato plants grown in stone wool slabs. There was no significant block effect for the different root and shoot parameters over all plants between the replicate experiments and therefore plants from the same treatments in both experiments were considered as individual samples (*n* = 10). The effect of treatment on root length values measured at different times points, namely at 7, 10, 14, and 21 days, determined by one-way ANOVA, was only temporal (Fig. 1). There were no significant treatment effects at days 7, 10, and 21, but there was a significant effect at day 14 (*P* = .013). Highest average root length values measured under treatments with strains E394 (128.6 cm), E390 (115.6 cm), C20 (114.6 cm), and E353 (113.5 cm) at day 14 significantly differed from all others. Comparisons between individual microbial treatments with respective controls at all four time points, by using Students *t*-test, revealed significant differences in average root length values at four occasions. Average root length values were higher and significantly different between treatment with strain E353 at day 10 (69.1 cm; *P* = .015) and between treatments with strains E394 (178.3 cm; *P* = .007) and E353 (182.7 cm; *P* = .04) at day 14 to corresponding controls (respectively, 24.3 and 90.2 cm). Treatment with strain FZB42 resulted in lower and significantly different average root length (71.3 cm; *P* = .019) than corresponding control (431.3 cm) at day 21. The effect of treatment on the number of adventitious roots at day 21, as determined by ANOVA, was significant at the level of *P* = .035. Treatments with strains FZB42, D5/23, CHC8, and Trianum-P resulted in lower number of adventitious roots than all other treatments and average values under these four treatments significantly differed from all others. No significant differences in average number of adventitious roots were present between individual microbial treatments and control as determined by Students *t*-test. The largest differences in total root length and number of adventitious roots at day 21 were in both cases between the treatments with strain FZB42 (respectively, 249.5 cm; 11.0 adventitious roots) and R043 (respectively 542.5 cm; 27.1 adventitious roots) (Fig. 1).

**Figure 2.**
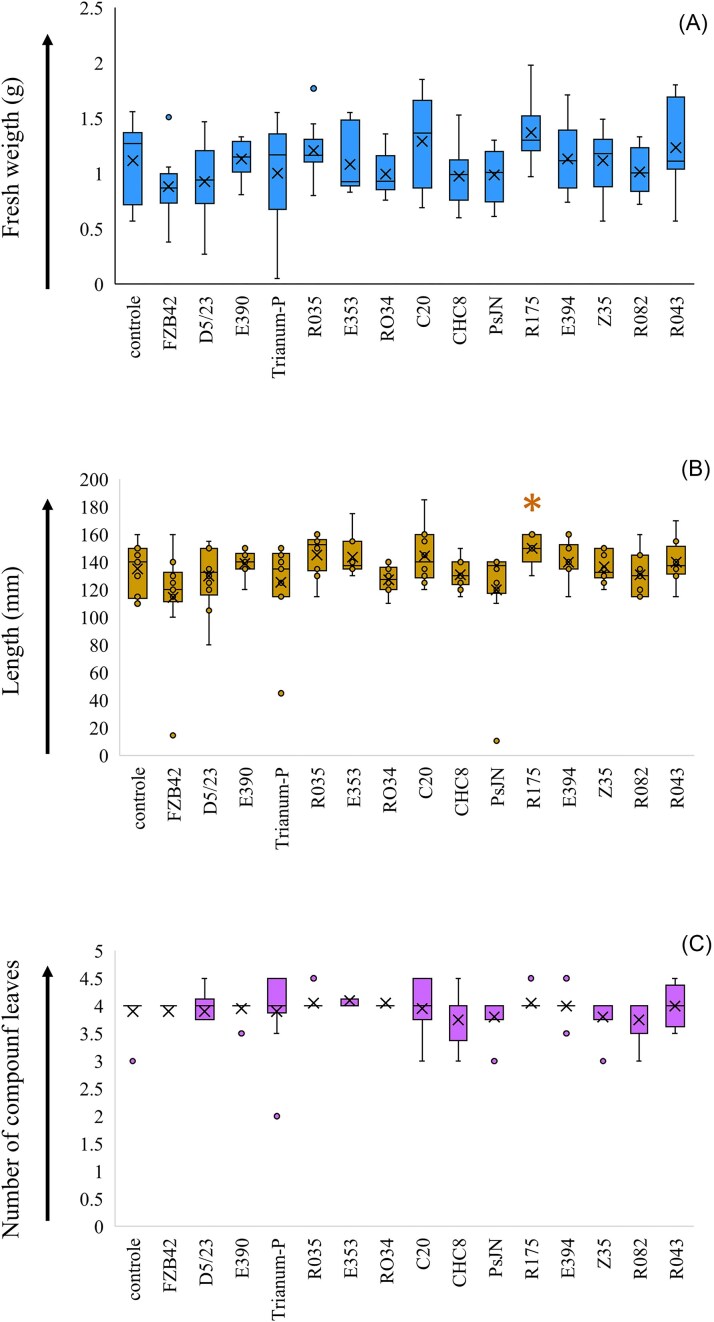
Box plot (maximum, 3rd, 2nd, and 1st quartiles, minimum values) of shoot fresh weight (A) and length values (B) and number of compound leaves (C) of tomato plants (*n* = 10) grown in stone wool slabs and sampled after 21 days. Box plot with * indicate significant different average value from control, as determined by Students *t*-test.

There were significant treatment effects on shoot length (*P* = .014) and fresh weight (*P* = .037) values after 21 d, but not on the number of compound leaves per plant, as calculated by one-way ANOVA. Largest differences in shoot length and fresh weight values were between treatments with strain FZB42 (on average 11.4 cm, 0.88 g) and strain R175 (on average 15.0 cm, 1.37 g) (Fig. 2). Differences in average shoot length values were significant (*P* = .046) between strain R175 and control (13.5 cm) as determined by Students *t*-test. No significant differences were further found between individual microbial treatments with respective controls for shoot fresh weight values and the number of compound leaves. Shoot length and fresh weight values and the number of compound leaves correlated with each other at a significance level of *P* < .001 [percentage variance accounted for (PVA), was between 28.7–55.9] as calculated by linear regression. The correlation between root length values and the number of adventitious roots was also significant (*P* < .001; PVA 25.0%). Comparisons between root and shoot parameters measured at day 21 revealed that correlations were occasionally present. Shoot length significantly correlated with root length at the level of *P* = .035 (PVA, 2.3%) and with the number of adventitious roots at the level of *P* = .016 (PVA, 3.2%). Shoot fresh weight significantly (*P* = .004; PVA, 4.6%) correlated with the number of adventitious roots and just above the level of significance (*P* = .067; PVA, 1.5%) with root length. There were no significant correlations between the number of compound leaves and root length values or number of adventitious roots.

### Effect of microbial treatments on differential gene expression in tomato plants grown in stone wool slabs

**Figure 3.**
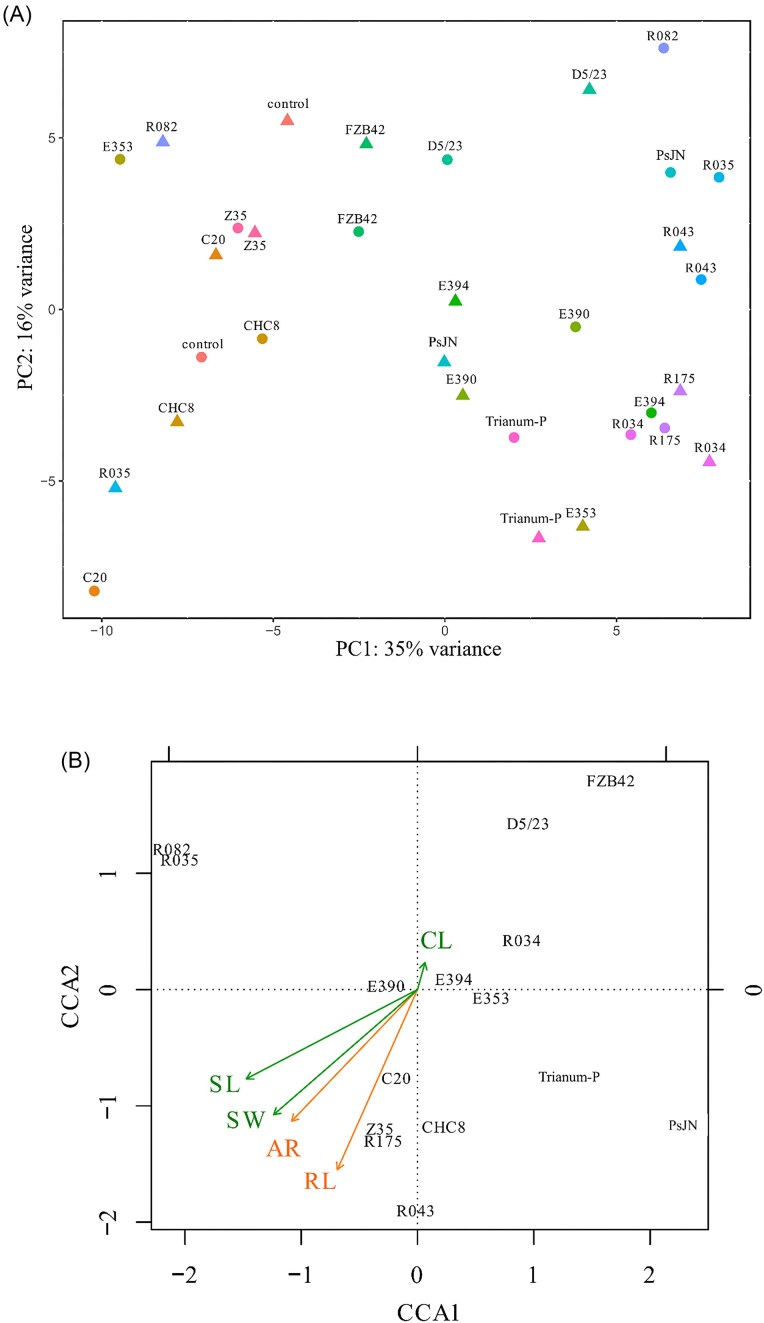
Principle component analysis biplot (A), demonstrating differences between gene expression profiles of tomato plants under 16 microbial and control treatments, and CCA biplot (B), demonstrating the relationship between gene expression profiles and shoot (CL, SL, SW) and root (AR, RL) parameters: CL, number of compound leaves; SL, shoot length; SW, shoot fresh weight; AR, number of adventitious roots; and RL, root length.

A total of 177 427 sequence reads were obtained from reverse transcribed RNA fragments from the 32 pooled stem samples of treated tomato plants grown for 21 d in stone wool slabs in two separate experiments. Expression profiles substantially differed between treatments, as determined by PCA (Fig. 3). Most of the variation (35%) was explained by differences in microbial and control treatments on the first axis in the ordination diagram. In 13 cases, replicate samples of same treatment were closely located from each other in the biplot, whereas in three treatments, with strains E353, R035, and R082, the individual samples were more distantly located from each other. This indicates that for the majority of the microbial and control treatments, the effects on tomato plant gene expression was congruous. When gene expression profiles of all 16 treatments were combined with shoot (fresh weight, length, and number of compound leaves) and root (length at day 21 and number of adventitious roots) parameters, included as metadata set in CCA (Fig. 3), it revealed that four treatments (R043, R175, Z35, and CHC8) positively correlated with both root, and with two of the three shoot (fresh weight and length) parameters. This in contrast to treatments with FZB42 and D5/23, of which the transcription profiles were located opposite of these four parameters, in the direction of the vector representing ‘number of compound leaves’ in the ordination diagram.

Copy numbers of individual transcripts from tomato stems under all 15 microbial treatments were compared with the ones from control plants and differences in number of transcripts varied per microbial treatment between 21 933 (treatment with strain R082) and 31 440 (strain PsJN) (Table S3). Of these transcripts, between 0 (strain E390) and 186 (strain CHC8) were Log2-fold higher or lower, and between 0 (strain E390) and 543 (strain CHC8) were significantly higher or lower in copy number under microbial than under control treatments. The number of transcripts that were both Log2-fold higher or lower and significantly different from control were between 0 (strain E390) and 165 (strain CHC8) and their transcribed genes are further denoted as differentially expressed genes (DEGs).

Genes that were differentially expressed in individual samples, among all 15 microbial treatments where highest root lengths to control were recorded at day 21, were selected. This resulted in a total of 11 genes that were increased, and 10 that were decreased in differential expression in these samples (Table 2). Expression of these 21 DEGs as potential ‘indicators of root growth’ was further evaluated in phase 2. Four microbial strains were selected for this evaluation, namely strains R043, Z35, FZB42, and T-22 (Trianum-P). Strains R043 and Z35 were selected because of their correlation with high root and shoot growth and strains FZB42 and T-22, in Trianum-P, were used as references. In opposite to strains R043 and Z35, strain FZB42 was correlated with low root and shoot growth (Fig. 3). From these four strains it was shown that they induced different genes upon treatments of tomato plants in phase 1 (Table S3).

**Table 2.** Selected transcripts that were differentially expressed in microbially treated samples, where highest root growth to control was measured in stone wool slabs at day 21. Quantitative primer sets were designed based on RNA sequences of these transcripts (Table S2).

Transcript number	Locus	Correlation to high or low root growth	Log_2_-fold change	Function
1	Solyc01g005305.1	High	5.7	Eukaryotic aspartyl protease family protein
2	Solyc01g091600.1	Low	−3.15	RING/Ubiquitin box superfamily protein
3	Solyc01g109160.4	Low	−1.01	Cytochrome P450 CYP74C4
4	Solyc02g084850.3	High	3.59	Abscisic acid/stress-inducible protein TAS14
5	Solyc03g115230.3	Low	−2.49	*S. lycopersicum* heat shock protein
6	Solyc03g115760.3	High	3.26	Vesicle transport SNARE protein
7	Solyc03g124110.2	High	1.02	C-repeat/ DRE binding factor (CBF)
8	Solyc05g010250.2	High	3.68	*N*-acetyltransferase
9	Solyc06g005990.3	High	7.25	Helicase
10	Solyc06g051650.1	High	3.5	Peptidyl-prolyl cis–trans isomerase
11	Solyc06g076780.3	Low	−1.3	Glycosyl hydrolase family protein
12	Solyc07g043580.3	High	1.02	Basic helix-loop-helix transcription factor 52
13	Solyc07g064160.3	High	3.66	Thiamine thiazole synthase
14	Solyc08g061500.2	Low	−3.04	Myosin
15	Solyc08g078470.3	Low	−2.81	Forkhead-associated domain-containing protein
16	Solyc09g011080.3	Low	−1.03	Ribulose bisphosphate carboxylase/oxygenase activase
17	Solyc09g065850.3	High	2.83	Auxin-regulated IAA3
18	Solyc10g078930.2	High	4.1	Activator of 90 kDa heat shock ATPase
19	Solyc10g081170.1	Low	3.04	Calmodulin 2
20	Solyc10g083450.2	Low	−2.98	NAC domain protein
21	Solyc12g013850.2	Low	−2.39	Core-2/I-branching beta-1

### Effect of four selected microbial treatments on plant development in stone wool blocks

Root systems of tomato plants under five microbial and control treatments were visualized by microtomography in dehydrated stone wool blocks (Fig. S2). A maximum growth period of 17 d was chosen to avoid roots growing outside the blocks. Namely, preliminary studies showed that longer incubation times led to root accumulation beneath the blocks, distorting accurate root size measurements by X-ray microtomography. Three of the applied microbial strains were recovered from the roots. Strain T-22 mycelium grew out on PDA agar from 17 day-old roots from plants treated with Trianum-P, but not from roots of control plants. Colonies identical in morphology to strain R043 were recovered from treated roots by plating, whereas these colonies were not recovered from roots of control plants. Furthermore, qPCRs specific for strains R043 and Z35 on root DNA extracts revealed 11–12 units lower average Ct values to control and differences were in both cases significant at levels of *P* < .001. Strain FZB42 was not detectable in roots, neither by plating, nor by qPCR, by making use of *B. velezensis*-specific primers, perfectly matching with the strain FZB42 genome sequence. The limit of detection in qPCR was established at 10 fg µl^−1^, corresponding to ~2.5 × 10^3^ FZB42 cell equivalents ml^−1^, and it can therefore not be excluded that FZB42 cells were present in, or near roots at undetectable levels.

**Figure 4.**
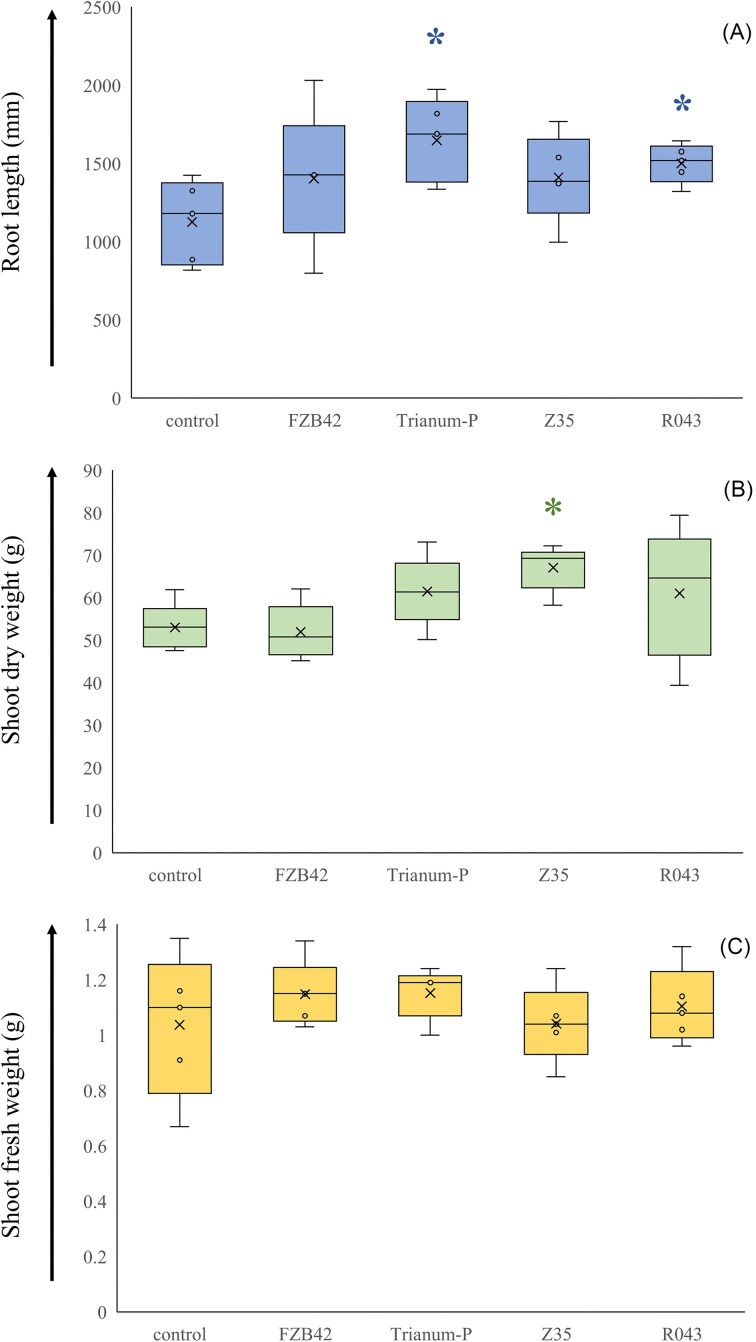
Box plot (maximum, 3rd, 2nd, and 1st quartiles, minimum values) of root length (A), shoot dry (B), and shoot fresh weight (C) values of tomato plants grown in stone wool blocks (*n* = 5) measured after 17 d under five different microbial and control treatments. Root length was determined by X-ray microtomography. Box plot with * indicate significant different average value from control, as determined by Student *t*-test.

There were no significant effects of treatment on root length and on shoot dry and fresh weight values of tomato plants grown in stone wool blocks as calculated by one-way ANOVA. Average root length values under treatments with Trianum-P (1649 mm) and strain R043 (1501 mm) were higher than that of control (1127 mm) and differences were in both cases significant at levels of, respectively, *P* = .015 and .022, as determined by Students *t*-test (Fig. 4). The average shoot dry weight value under treatment with strain Z35 (67.1 mg) was higher than control (53.0 mg) and this difference was significant at the level of *P* = .004. Furthermore, there were positive correlations between root length and shoot dry (*P* = .066; PVA, 10.2) and fresh weight (*P* = .009; PVA, 23.1%) values, as calculated by linear regression. When average shoot fresh weight values were compared between tomato plants grown in stone wool blocks and in slabs at day 21, then overall values were not significantly different between plants grown in blocks and in slabs (difference of 1.04) as calculated by Students *t*-test. However, when average root length values of plants under the same treatments were compared, then averages significantly (*P* < .001) differed from each other and the average root size in blocks was five times higher than in slabs.

### Quantitative PCR on cDNA samples from treated tomato plants grown in stone wool blocks

Ct values of the qPCRs with 21 primer sets on RNA extracts from all 25 tomato plants varied between 23.7 and not detectable (>40). Nondetectable Ct values were replaced for 40, to a maximum of three, for the primer set targeting transcript 17 (further denoted as primer set 17, Table S2) only, whereas numbers of nondetectable Ct values were higher for the primer sets 4, 5, 10, and 20 and Ct values from these primer sets were excluded from further statistical analyses.

The effect of microbial and control treatments on Ct values was significant for primer sets 13 (*P* < .001) and 18 (*P* = .002) and just above the arbitrary level of significance for 17 (*P* = .059), as calculated by one-way ANOVA (Table S4). Highest Ct values of primer sets 13 and 18 were found for treatment with strain FZB42 and differences with the other four microbial and control treatments were significant, indicating that both targeted transcripts were lower under treatment with strain FZB42 than under the other treatments. Significant negative correlations of Ct values with root length was observed for primer set 8 (*P* = .029; PVA, 15.7) and for primer set 16 (*P* = .029; PVA, 15.6). Furthermore, Ct values showed significant negative correlations with shoot fresh weight values for primer set 8 (*P* < .001; PVA, 41.4) and with shoot dry weight values for primer sets 8 (*P* = .026; PVA, 16.3) and 18 (*P* = .018; PVA, 18.7), as determined by linear regression (Table S4).

Significant correlations were observed among the different primer sets (Table S5). Strongest correlation was found between primer sets 1, 13, 16, and 18 with *P*-values between .008 and <.001 (PVA between 23.8 and 80.4). Primer sets 12 and 19 occasionally correlated with primer sets 1, 13, 16, and 18. Weaker correlations were found between primer sets 2, 7, and 9 [*P*-values of .019 between 2 and 7 (PVA, 18.4) and of 0.03 between primers 7 and 9 (PVA, 15.3)]. A total of nine genes were thus coexpressed under the experimental circumstances, and the transcripts of other eight genes (3, 6, 8, 11, 14, 15, 17, and 21) not. In summary, there were positive correlations between the copy number of transcript 8, coding for a *N*-acetyltransferase (Table 2), and root length and shoot fresh and dry weight values, and between the copy number of transcript 16, coding for a ribulose biphosphate carboxylase/oxygenase (Rubisco) activase, and root length values. Expression of transcript 8 was independent of any of the other evaluated transcripts under the experimental circumstances, whereas that of transcript 16 was coexpressed with three other transcripts.

## Discussion

Selection of transcripts reporting on root growth upon microbial treatments at the early tomato plant growth stage was endeavored in this study. Strain selection was based on association with plants and diversity in taxonomic affiliation. Strains PsJN, D5/23, R034, R035, R043, R082, and R175 are endophytes derived from monocotyledonous (onion, wheat, and rice), and strains E353, E390, and E394 from dicotyledonous (tomato) plants. The three *Verrucomicrobium* subdivision 1 strains CHC8, C20, and Z35 are not endophytes, but inhabitants of leek and potato rhizosphere soils (Nunes da Rocha et al. 2010, 2011). Although their exact modes of action in tomato plants were unknown for the majority of the strains, we anticipated, based on their close associations with plants, that these strains would have different effects on root development, shoot growth and gene expression. Altogether, microbial treatments resulted in strong effects on tomato root growth and effects were stronger on roots than on shoots. Further, microbial inoculations resulted in a wide diversity in gene expression profiles, enabling us to select 21 transcripts as potential indicators of early root growth.

Root measurements in stone wool blocks by X-ray microtomography was more accurate than in slabs by visible inspection. This was clear from the five times higher root length measured in stone wool blocks than in slabs. This difference in measured root sizes can be explained by the fact that not all roots in slabs were visible by eye, but also that roots were more limited in growth by the available volume that was smaller in slabs than in blocks. Shoot sizes of the tomato plants were, to the contrary, about the same under the two growth circumstances, indicating that shoot growth was not, or to a much lesser extent, affected by cultivation in slabs. The period of growth of tomato plants in stone wool slabs and blocks was relatively short because of experimental constrains with respect to outgrowth of roots from stone wool slabs and blocks. Our observations on root and shoot growth and gene expression was therefore limited to the young plant growth stage. The experimental period is relevant for practical applications because microbial treatments often will be applied at the nursery stages of plant cultivation and development of roots is often critical in this particular plant growth stage.

Variation in differential gene expression under the different microbial treatments was striking, especially with respect to treatments with the three strains belonging to *Verrucomicrobium* subdivision 1 clade, i.e. C20, CHC8, and Z35. Highest DEG numbers were recorded for treatments with these strains, whereas numbers were occasionally lower and even zero for other treatments. The three *Verrucomicrobium* subdivision 1 strains belonged to different taxonomical clades within subdivision 1, namely to *Luteolibacter* (strain C20) and to a new group tentatively named as *Candidatus* genus *Rhizospheria* (strains CHC8 and Z35) (Nunes da Rocha et al. 2010, 2011). It was already known that all three strains could live in association with plants and that they possessed some root growth stimulatory activities (Nunes et al. 2011). A total of 145 genes were differentially expressed upon tomato plant treatment with strain Z35, of which 13 were selected for further screening because differential expression of these genes was associated with high root growth in comparison to control. Among these 13 DEGs, two were involved in hormone regulation, i.e. abscisic acid/stress-inducible protein TAS14 and an auxin-regulated IAA3 gene, indicating that strain Z35 can influence plant growth and development via different hormone-regulated pathways. Furthermore, strain Z35 colonized roots grown in stone wool blocks, which makes clear that this strain is an avid root colonizer of tomato plants. Altogether, this sheds new light on the mode of action of representatives of *Verrucomicrobium* subdivision 1. Our results confirm observations made in other studies wherein it was shown that *Verrucomicrobium* strains interact with plants (Nunes da Rocha et al. 2011, Ranjan et al. 2015, Bünger et al. 2020). Therefore, it can be concluded that particular groups within the phylum of *Verrucomicrobiota* interact with plants without causing them any visible harm and therefore these groups must be considered as plant-associated rhizobacteria.

Two other strains used for tomato plant treatments in stone wool blocks were shown to colonize plants, namely T-22, as active component of Trianum-P, and R043. Strain T-22 is known to stimulate plant development and to induce resistance in tomato plants (Vitti et al. 2016). Strain R043 possesses IAA and ACC deaminase genes (Hardoim et al. 2011), indicating that this strain has the potential to interfere in hormone regulation in plants, but it is unknown whether these genes are expressed under the applied circumstances. Because strain R043 originates from rice, it demonstrates its rather divers plant host spectrum. Strain FZB42 was not recoverable from tomato roots, but considering the 62 DEGs detected in strain FZB42-treated tomato plants and the significantly reduced expression of two genes encoding thiamine thiazole synthase and activator of 90 kDa heat shock ATPase, it is clear that this strain must have had an interaction with tomato plants under the experimental circumstances. Possibly, strain FZB42 need a short contact time with tomato plants for specific gene induction. In studies performed by Fan et al. (2012) and Chowdhury et al. (2015), it was demonstrated that strain FZB42 colonized *Arabidopsis thaliana*, lettuce, and maize roots, but that it was not a strong competitor in all of these plant species. Strain FZB42 produce auxin in duckweed (Idris et al. 2007), indicating that this strain can influence auxin-regulated pathways in planta. The observed decrease in root and shoot growth upon treatment with strain FZB42, compared to treatments with the other strains and with the untreated control, makes clear that gene induction in tomato plants by strain FZB42 initially leads to growth retardation. Since strain FZB42 is described as plant growth-promoting rhizobacterium (Fan et al. 2012), it is possible that it induces systemic resistance pathways in tomato plants upon inoculation. This is a resource and energy consuming process for plants that initially results in growth retardation and at later stages of growth to increased resilience to different forms of stress (Hoang et al. 2020).

Seventeen of the 21 evaluated primer sets applied on reverse transcribed RNA samples from tomato plants grown in stone wool blocks gave reliable Ct values and this indicates that expression of most of the selected genes was detectable in individual plants cultivated under these circumstances. That four primer sets did not give reliable Ct values may rely on the fact that composite samples from five plants were used for RNAseq analysis on tomato plants grown in slabs, whereas qPCR analysis was performed on individual plants grown in blocks. The correlations between Ct values from primer set 8, targeting a *N*-acetyltransferase gene, and from 16, targeting a Rubisco activase gene, with root lengths values, and for primer set 8 also with shoot fresh and dry weight values, indicate that both targeted transcripts can be a good indicators for root development upon microbial treatments. Under the evaluated circumstances, the *N*-acetyltransferase gene was expressed independent of any of the other transcripts whereas the Rubisco activase gene was coexpressed with three other genes encoding for an eukaryotic aspartyl protease family protein, thiamine thiazole synthase, and activator of 90 kDa heat shock ATPase. Translated products of these three genes are known to be involved in heat and drought stress tolerances and in plant growth and development (Salvucci 2008, Cao et al. 2019, Yin et al. 2022). Coexpression of these genes is illustrative for the regulatory network controlling the balance between growth and stress tolerance in plants (Hoang et al. 2020). Most likely, the introduced microbes interfere with this regulatory network in tomato plants.

*N*-acetyltransferases are commonly involved in plant development and resilience to stresses. It was shown that *N*-terminal acetyltransferase regulate plant growth and protection against osmotic stress in Arabidopsis (Feng et al. 2020) and histone acetyltransferase in development of flower meristem in tomato plants (Hawar et al. 2022). However, the targeted gene (Solyc05g010250) is annotated as a chloroplast-localized acetyltransferase (*slsnat2*), member of the general control nonrepressible 5-related *N*-acetyltransferases superfamily (Wang et al. 2022). Its gene product, SlSNAT2, catalyze lys acetylation of the large ribulose-1,5-biphosphate carboxylase/ oxygenase (Rubisco) subunit and negatively regulates drought tolerance in tomato plants (Wang et al. 2022). Ribulose biphosphate carboxylase/oxygenase activase (Rca) regulates Rubisco activity by adenosine triphosphate-dependent conformational changes in Rubisco protein folding (Amaral et al. 2024). It is a remarkable fact that we identified two genes that are both involved in modulation of Rubisco activity via our screening on microbial-stimulated root growth. Most likely genes expressing enzymes involved in the modulation of Rubisco activity are appropriate candidates as indicators of root growth in general upon microbial treatments. Regulation of Rubisco activity is controlled under different environmental conditions (Amaral et al. 2024) and plant-associated microorganisms may play a role in induction of Rubisco activity regulation. The exact link between regulation of Rubisco activity and stimulation of root growth is not clear and needs further investigation. It, however, creates new opportunities to select for targets in plants that report on increased root and shoot growth, but possibly also on tolerance to abiotic stresses upon microbial treatments (Wang et al. 2022).

## Conclusion

Two genes that were differentially expressed upon stimulated root growth by microbial treatments were identified. Both genes are involved in regulation of Rubisco activity and their transcripts are indicators for root development at the early plant growth stage. These indicators can be applied in high throughput screenings of plants treated with different microbes or consortia of microbes for preselection of root growth stimulants. More accurate screenings can be applied in a follow up stage with a selection of microbial strains in a comparable way as performed in this study. Final selections of microbial strains can be used as active ingredients in microbial products and these products are valuable for applications as plant growth stimulants in sustainable crop production.

## Supplementary Material

fiaf063_Supplemental_Files
