# Supplementary material for: The quest for molecular markers indicating root growth in microbially treated tomato (Solanum lycopersicum) plants
Source: FEMS Microbiol Ecol. 2025 Jun 18;101(7):fiaf063. doi: 10.1093/femsec/fiaf063 (PMC12199702; doi:10.1093/femsec/fiaf063)
Supplement: fiaf063_Supplemental_Files [file fiaf063_supplemental_files.zip › Supp info.docx]

**
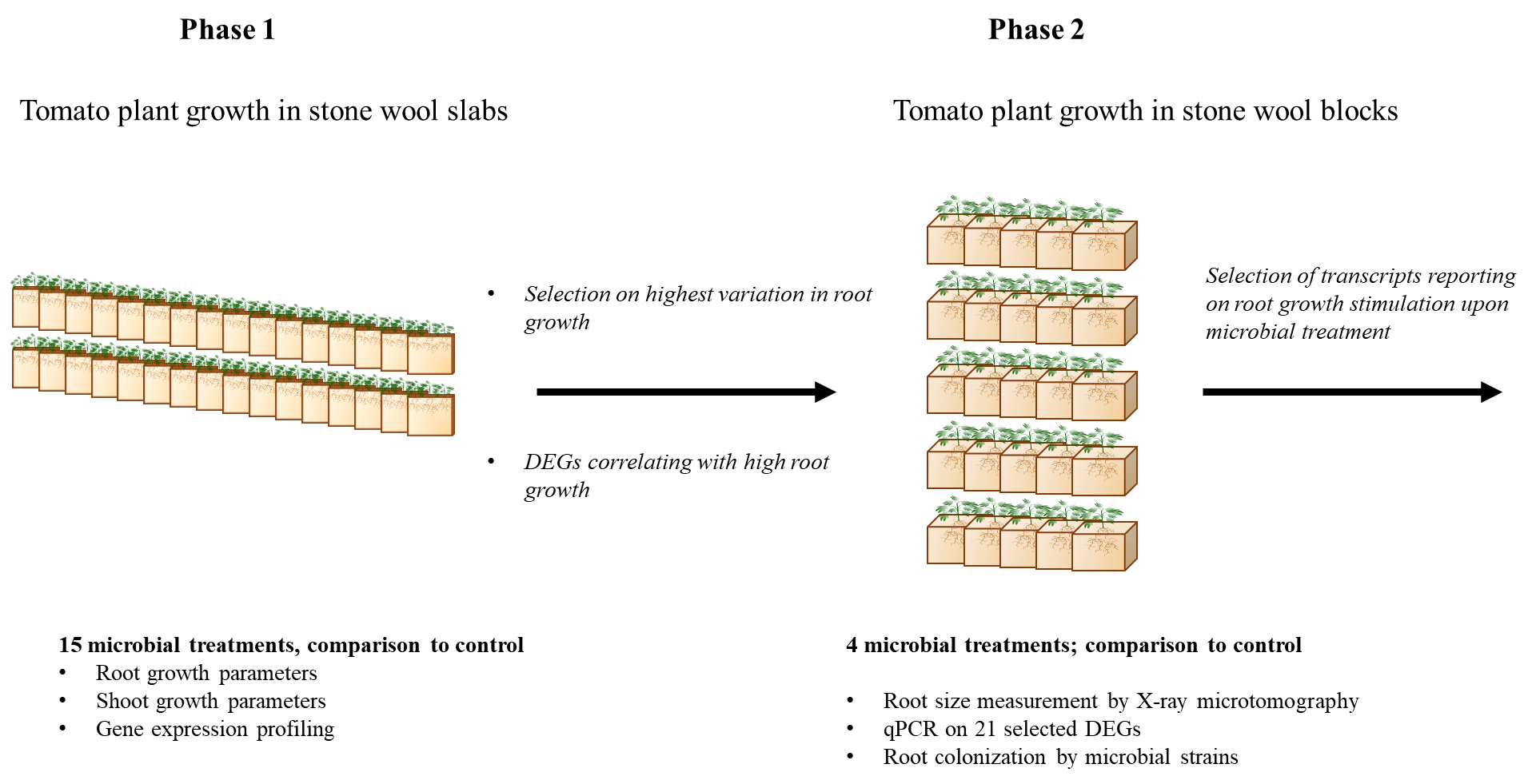
**

**Fig. S1**. Experimental design of the study.

**
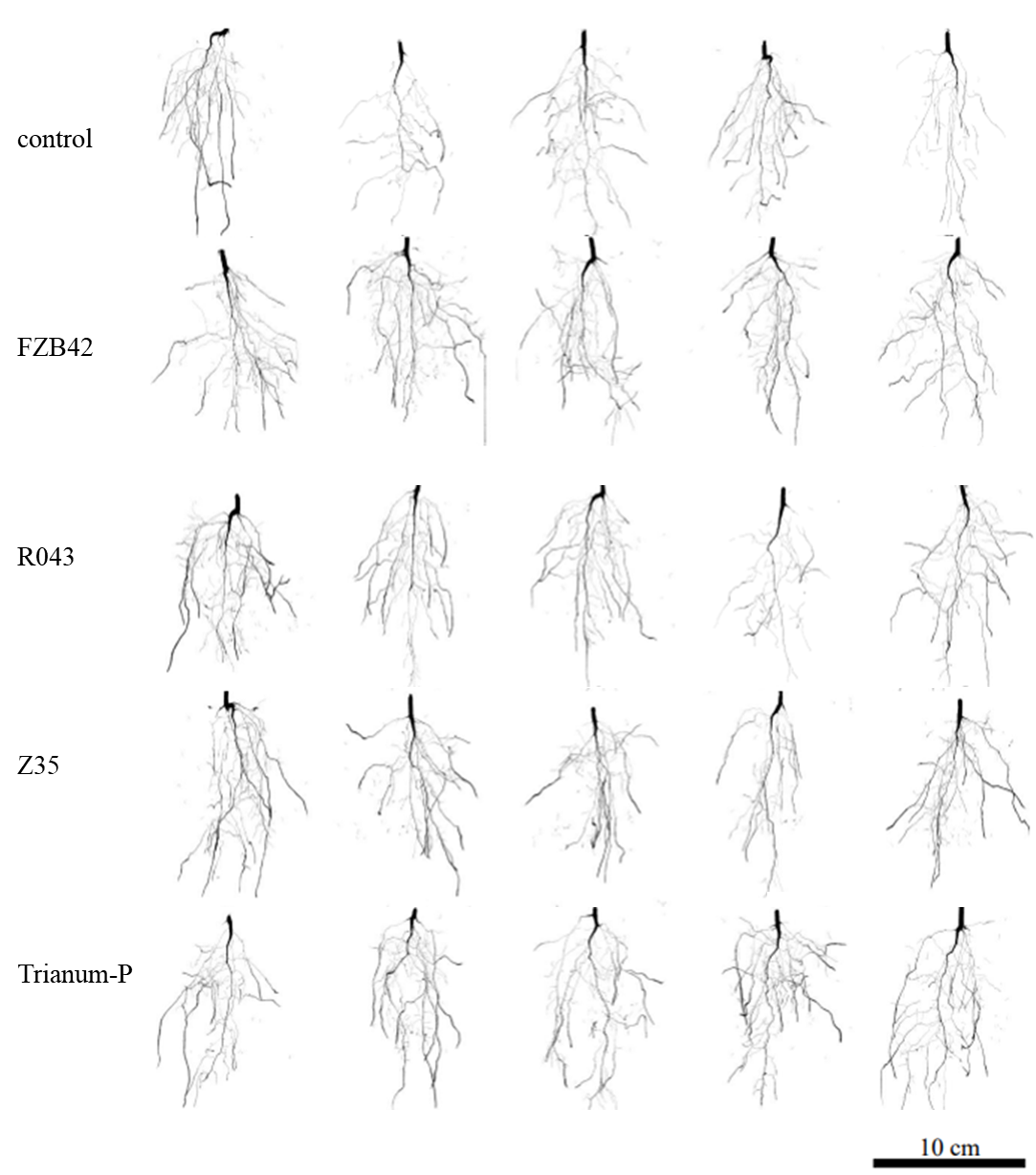
**

**Fig. S2**. Root systems, measured in 3 dimensions using X-ray microtomography, of tomato plants grown in stone wool blocks under five different microbial and control treatments. The 2D XZ view is shown in this figure.

**Table S1.** Primers sets used for detection of bacterial strains in inoculated tomato plants grown in stone wool blocks

| Strain | Primer name | Sequence (5’→3’) | Origin / reference |
| --- | --- | --- | --- |
| Z35 | VS1Bf | GCCCGACAGGGTTGATAGTA | Nunes da Rocha et al. 2010 |
|  | VS1Br | CGCTTGGGACCTTCGTATTA |  |
| FZB42 | Bamy1F | AAGGCAAGCCTAGCGAAAAT | Rotolo et al. 2016 |
|  | Bamy1R | CACATCGCTGTGCGATTTAT |  |
| R043 | R043_F_long | ACTCGAACAGGGTGCTAACG | This study |
|  | R043_R_long | TAGCTCACCATCTCCTCCGC |  |

References:

Nunes da Rocha, U., Andreote, F.D., de Azevedo, J.L., van Elsas, J.D., and van Overbeek, L.S. 2010. Cultivation of hitherto-uncultured bacteria belonging to the *Verrucomicrobia* subdivision 1 from the potato (*Solanum tuberosum* L.) rhizosphere. J. Soils Sed. 10: 326–339. [doi.org/10.1007/s11368-009-0160-3](https://doi.org/10.1007/s11368-009-0160-3)

Rotolo, C., De Miccolis Angelini, R.M., Pollastro, S., and Faretra, F. 2015. A TaqMan-based qPCR assay for quantitative detection of the biocontrol agents *Bacillus subtilis* strain QST713 and *Bacillus amyloliquefaciens* subsp. *plantarum strain* D747. BioControl 61: 91–101. doi.org/10.1007/s10526-015-9701-4

| **Name** | **Locus** | **Forward (5’ → 3’)** | **Reverse (5’ → 3’)** | **Amplicon size (bp)** |
| --- | --- | --- | --- | --- |
| Set1 | Solyc01g005305.1 | GTCATTAAGCAATCCCTGTCTCC | GGTGGAGATGATTTGACACTCAC | 111 |
| Set2 | Solyc01g091600.1 | GAATTCAGCTTAGCGATTCATCTCC | GGTGAAACTGGTGATTATCAGTCTG | 117 |
| Set3 | Solyc01g109160.4 | GTCTTCCAGTTGGAGAAATCCC | GGCACAGACGAATTCTTCCGTA | 108 |
| Set4 | Solyc02g084850.3 | GCACAATACGGCAATCAAGACC | GGAGTCTATCAAGGTACCGGTA | 88 |
| Set5 | Solyc03g115230.3 | GATACATTACAGGTCGACATTTGCC | GGAAGAGGATTCAGCTAGAAGTTGA | 139 |
| Set6 | Solyc03g115760.3 | GAGTTTACCTCCAAATGTTAAAGCC | GGGATGAATTGTTGGAATCGGGAAT | 147 |
| Set7 | Solyc03g124110.2 | AGAAGTTTCGAGAAACTCGACATCC | GGGTTTGTGAAGTCAGAGAACCAAA | 88 |
| Set8 | Solyc05g010250.2 | AATCCAAATCGCATTAGAGAACACC | GGCGTATTCAATGCGATTATTTGGG | 119 |
| Set9 | Solyc06g005990.3 | AAATCCTTCCAGATCCATCTTTGCC | GGGACAGATTGGATAATGGAAGATG | 97 |
| Set10 | Solyc06g051650.1 | AGCAGTTTCCACAGGATCATACC | GGCGAGGTGGAGAGTCAATATAT | 87 |
| Set11 | Solyc06g076780.3 | AGATCGAAAGAGCTGTTGCTAATCC | GGAAGTAGACCATTTGAGAATGCAG | 96 |
| Set12 | Solyc07g043580.3 | CTATCAACGCCATTTGCAGAATCC | GGTTCACATACAGCACAGCAAACT | 130 |
| Set13 | Solyc07g064160.3 | CAAACACGCTGCCTTGTTCACC | GGAAGAGTCGGTGGTGTTGTCA | 128 |
| Set14 | Solyc08g061500.2 | GAACAACAAGTTCTCGAGTCTAACC | GGTAGAATATCTGGAGCTGCAATTC | 142 |
| Set15 | Solyc08g078470.3 | TATTGCAATTGACCCATCTCGACC | GGAAGAAATCGAGGTTAAGGGTAG | 135 |
| Set16 | Solyc09g011080.3 | TTGTCAAGATCGTCGATAGCTTCC | GGCGAGAGTATACGATGATGAAGT | 82 |
| Set17 | Solyc09g065850.3 | AGGATATAATGGATCTGATTATGCACC | GGCTTAGAATTATCAAAGGATCTGAAG | 131 |
| Set18 | Solyc10g078930.2 | GTCACGAAACTTCCTCACTAACC | GGGAAGATTATTCCGGGTTACGA | 147 |
| Set19 | Solyc10g081170.1 | AGCTTCGTCATGTGATGACTAACC | GGAGTTTGTTAAGGTCATGATGGC | 130 |
| Set20 | Solyc10g083450.2 | GCTACCGGAGCTGACAAACC | GGGTAAATGAGTCTCGCAGAATC | 147 |
| Set21 | Solyc12g013850.2 | CAGCATCCACATACACTTACCC | GGAGTAATGGTAGCTTCACACC | 146 |

**Table S2.** Primer sets used for detection of 21 differentially expressed genes reporting on root growth.

| Treatment with strain |  | Total number of transcripts expressed | Number of transcripts that are Log2-fold higher or lower than control | Number of transcripts per treatment that are significantly (P≤0.05) different to control | Number of DEGs |
| --- | --- | --- | --- | --- | --- |
| **T22** |  | 22282 | 3 | 19 | 3 |
| **FZB42** |  | 27460 | 62 | 431 | 62 |
| PJSN |  | 31440 | 31 | 84 | 31 |
| D5/23 |  | 28425 | 61 | 437 | 61 |
| R034 |  | 28035 | 116 | 447 | 116 |
| R035 |  | 21986 | 11 | 3 | 3 |
| **R043** |  | 25203 | 53 | 184 | 53 |
| R082 |  | 21933 | 15 | 3 | 3 |
| R175 |  | 24100 | 50 | 170 | 50 |
| E353 |  | 23555 | 103 | 487 | 102 |
| E390 |  | 23084 | 0 | 0 | 0 |
| E394 |  | 24412 | 151 | 457 | 131 |
| CHC8 |  | 22728 | 186 | 543 | 165 |
| C20 |  | 29968 | 112 | 368 | 107 |
| **Z35** |  | 24585 | 164 | 434 | 145 |

**Table S3**. Number of differentially expressed genes (DEGs) in tomato stems upon individual treatments with 15 microbial strains to control (A), number of DEGs that are exclusive and overlapping between four selected strains applied in phase 2 of the experiment with tomato plants grown in stone wool blocks (B) and presence (+) of selected transcripts among DEGs under treatment with each of the four selected microbial strains (C).

B.

|  | FZB42 | Trianum-P | Z35 | R043 |
| --- | --- | --- | --- | --- |
| total | 62 | 3 | 145 | 53 |
| exclusive | 43 | 1 | 96 | 6 |
| overlapping | 19 | 2 | 49 | 47 |
| %unique | 69.4% | 33.3% | 66.2% | 11.3% |

C.

| Transcript | FZB42 | Trianum-P | Z35 | R043 |
| --- | --- | --- | --- | --- |
| 1 |  |  | + | + |
| 2 |  |  |  |  |
| 3 |  |  |  |  |
| 4 |  |  | + |  |
| 5 |  |  | + | + |
| 6 |  |  | + |  |
| 7 |  |  |  |  |
| 8 |  |  | + | + |
| 9 |  |  | + | + |
| 10 |  |  |  |  |
| 11 |  |  |  |  |
| 12 |  |  |  |  |
| 13 |  |  | + | + |
| 14 |  |  | + |  |
| 15 |  |  | + |  |
| 16 |  |  |  |  |
| 17 |  |  | + | + |
| 18 |  |  | + | + |
| 19 |  |  |  |  |
| 20 |  |  | + |  |
| 21 |  |  | + |  |

| Root/ shoot parameters and primer set* | One-way ANOVA  (P value) | Linear regression | | | | | |
| --- | --- | --- | --- | --- | --- | --- | --- |
|  |  | RL | | SFW | | SDW | |
|  |  | P value | PVA† | P value | PVA | P value | PVA |
| RL | 0.119 |  |  | 0.009 | 23.1 | 0.066 | 10.2 |
| SFW | 0.671 |  |  |  |  | <0.001 | 53.6 |
| SDW | 0.074 |  |  |  |  |  |  |
| 1 | 0.192 | 0.589 | 0 | 0.932 | 0 | 0.781 | 0 |
| 2 | 0.775 | 0.226 | 2.2 | 0.391 | 0 | 0.156 | 4.6 |
| 3 | 0.716 | 0.606 | 0 | 0.446 | 0 | 0.88 | 0 |
| 6 | 0.676 | 0.354 | 0 | 0.062 | 10.6 | 0.349 | 0 |
| 7 | 0.713 | 0.625 | 0 | 0.7 | 0 | 0.525 | 0 |
| 8 | 0.7 | **0.029** | 15.7 | **<.001** | 41.4 | **0.026** | 16.3 |
| 9 | 0.596 | 0.426 | 0 | 0.617 | 0 | 0.615 | 0 |
| 11 | 0.835 | 0.493 | 0 | 0.233 | 2 | 0.824 | 0 |
| 12 | 0.526 | 0.243 | 1.8 | 0.349 | 0 | 0.083 | 8.7 |
| 13 | **<.001** | 0.872 | 0 | 0.959 | 0 | 0.114 | 0 |
| 14 | 0.17 | 0.587 | 0 | 0.982 | 0 | 0.168 | 4.1 |
| 15 | 0.972 | 0.731 | 0 | 0.136 | 5.4 | 0.104 | 7.2 |
| 16 | 0.172 | **0.029** | 15.6 | 0.528 | 0 | 0.409 | 0 |
| 17 | 0.059 | 0.981 | 0 | 0.928 | 0 | 0.941 | 0 |
| 18 | **0.002** | 0.43 | 0 | 0.328 | 0 | **0.018** | 18.7 |
| 19 | 0.103 | 0.902 | 0 | 0.754 | 0 | 0.555 | 0 |
| 21 | 0.556 | 0.632 | 0 | 0.288 | 0.8 | 0.353 | 0 |

**Table S4**. Effect or treatment on root length, shoot dry and fresh weight and Ct values and correlations between these parameters, calculated by, respectively, one-way ANOVA and linear regression.

- RL, root length; SFW, shoot fresh weight; SDW, shoot dry weight.
- PVA, Percentage variance accounted for

**Table S5.** P-values indicating significance in correlations between Ct values made with different primer sets (A) and the percentage variance accounted for (B) calculated by linear regression analysis.

| **A** | Set 1 | Set 2 | Set 3 | Set 6 | Set 7 | Set 8 | Set 9 | Set 11 | Set 12 | Set 13 | Set 14 | Set 15 | Set 16 | Set 17 | Set 18 | Set 19 | Set 21 |
| --- | --- | --- | --- | --- | --- | --- | --- | --- | --- | --- | --- | --- | --- | --- | --- | --- | --- |
| Set 1 |  | 0.211 | 0.202 | 0.185 | 0.564 | 0.629 | 0.55 | 0.92 | 0.68 | **0.005** | 0.36 | 0.735 | **<.001** | 0.755 | **0.008** | 0.92 | 0.318 |
| Set 2 |  |  | 0.226 | 0.598 | **0.03** | 0.32 | 0.077 | 0.248 | 0.355 | 0.892 | 0.141 | 0.101 | 0.83 | 0.434 | 0.37 | 0.406 | 0.956 |
| Set 3 |  |  |  | 0.744 | 0.172 | 0.723 | 0.687 | 0.733 | 0.553 | 0.553 | 0.579 | 0.415 | 0.24 | 0.74 | 0.726 | 0.825 | 0.624 |
| Set 6 |  |  |  |  | 0.25 | 0.071 | 0.918 | 0.08 | 0.702 | 0.415 | 0.616 | 0.07 | 0.496 | 0.194 | 0.571 | 0.245 | 0.92 |
| Set 7 |  |  |  |  |  | 0.885 | **0.019** | 0.573 | 0.46 | 0.996 | 0.146 | 0.766 | 0.602 | 0.497 | 0.98 | 0.538 | 0.581 |
| Set 8 |  |  |  |  |  |  | 0.314 | 0.749 | 0.463 | 0.505 | 0.906 | 0.95 | 0.409 | 0.836 | 0.749 | 0.582 | 0.554 |
| Set 9 |  |  |  |  |  |  |  | 0.551 | 0.582 | 0.699 | 0.229 | 0.237 | 0.632 | 0.154 | 0.683 | 0.523 | 0.074 |
| Set 11 |  |  |  |  |  |  |  |  | 0.864 | 0.882 | 0.688 | 0.582 | 0.423 | 0.962 | 0.837 | **0.003** | 0.889 |
| Set 12 |  |  |  |  |  |  |  |  |  | 0.18 | 0.514 | 0.591 | 0.068 | 0.502 | **0.026** | 0.323 | 0.357 |
| Set 13 |  |  |  |  |  |  |  |  |  |  | 0.923 | 0.499 | **0.006** | 0.58 | **<.001** | 0.926 | 0.284 |
| Set 14 |  |  |  |  |  |  |  |  |  |  |  | 0.463 | 0.15 | 0.593 | 0.89 | 0.169 | 0.344 |
| Set 15 |  |  |  |  |  |  |  |  |  |  |  |  | 0.689 | 0.296 | 0.338 | 0.298 | 0.247 |
| Set 16 |  |  |  |  |  |  |  |  |  |  |  |  |  | 0.213 | **<.001** | 0.908 | 0.333 |
| Set 17 |  |  |  |  |  |  |  |  |  |  |  |  |  |  | 0.408 | 0.424 | 0.962 |
| Set 18 |  |  |  |  |  |  |  |  |  |  |  |  |  |  |  | 0.831 | 0.09 |
| Set 19 |  |  |  |  |  |  |  |  |  |  |  |  |  |  |  |  | 0.738 |

|  |  |
| --- | --- |
|  | |

| **B** | Set 1 | Set 2 | Set 3 | Set 6 | Set 7 | Set 8 | Set 9 | Set 11 | Set 12 | Set 13 | Set 14 | Set 15 | Set 16 | Set 17 | Set 18 | Set 19 | Set 21 |
| --- | --- | --- | --- | --- | --- | --- | --- | --- | --- | --- | --- | --- | --- | --- | --- | --- | --- |
| Set 1 |  | 2.7 | 2.9 | 3.5 | 0 | 0 | 0 | 0 | 0 | 26.8 | 0 | 0 | 43.8 | 0 | 23.8 | 0 | 0.2 |
| Set 2 |  |  | 2.2 | 0 | 15.3 | 0.1 | 9.2 | 1.7 | 0 | 0 | 5.2 | 7.4 | 0 | 0 | 0 | 0 | 0 |
| Set 3 |  |  |  | 0 | 4.0 | 0 | 0 | 0 | 0 | 0 | 0 | 0 | 1.9 | 0 | 0 | 0 | 0 |
| Set 6 |  |  |  |  | 1.6 | 9.7 | 0 | 8.9 | 0 | 0 | 0 | 9.8 | 0 | 3.2 | 0 | 1.7 | 0 |
| Set 7 |  |  |  |  |  | 0 | 18.4 | 0 | 0 | 0 | 5.0 | 0 | 0 | 0 | 0 | 0 | 0 |
| Set 8 |  |  |  |  |  |  | 0.3 | 0 | 0 | 0 | 0 | 0 | 0 | 0 | 0 | 0 | 0 |
| Set 9 |  |  |  |  |  |  |  | 0 | 0 | 0 | 2.2 | 1.9 | 0 | 4.6 | 0 | 0 | 9.5 |
| Set 11 |  |  |  |  |  |  |  |  | 0 | 0 | 0 | 0 | 0 | 0 | 0 | 28.7 | 0 |
| Set 12 |  |  |  |  |  |  |  |  |  | 3.7 | 0 | 0 | 10.0 | 0 | 16.3 | 0.1 | 0 |
| Set 13 |  |  |  |  |  |  |  |  |  |  | 0 | 0 | 25.6 | 0 | 80.4 | 0 | 0.8 |
| Set 14 |  |  |  |  |  |  |  |  |  |  |  | 0 | 4.8 | 0 | 0 | 4.0 | 0 |
| Set 15 |  |  |  |  |  |  |  |  |  |  |  |  | 0 | 0.6 | 0 | 0.5 | 1.7 |
| Set 16 |  |  |  |  |  |  |  |  |  |  |  |  |  | 2.6 | 38.9 | 0 | 0 |
| Set 17 |  |  |  |  |  |  |  |  |  |  |  |  |  |  | 0 | 0 | 0 |
| Set 18 |  |  |  |  |  |  |  |  |  |  |  |  |  |  |  | 0 | 8.2 |
| Set 19 |  |  |  |  |  |  |  |  |  |  |  |  |  |  |  |  | 0 |
