# Supplementary figures and images for: The quest for molecular markers indicating root growth in microbially treated tomato (Solanum lycopersicum) plants
Source: FEMS Microbiol Ecol. 2025 Jun 18;101(7):fiaf063. doi: 10.1093/femsec/fiaf063 (PMC12199702; doi:10.1093/femsec/fiaf063)

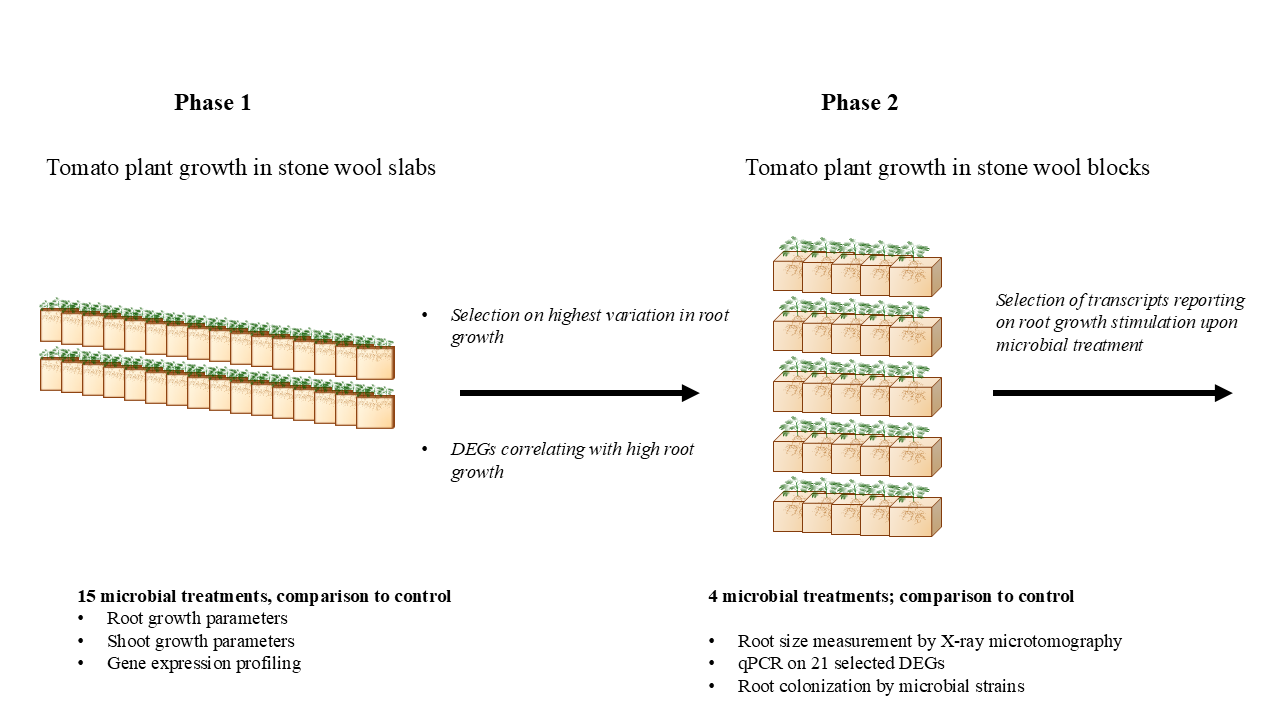

Supplement: fiaf063_Supplemental_Files [file fiaf063_supplemental_files.zip › Fig S1 .tif]

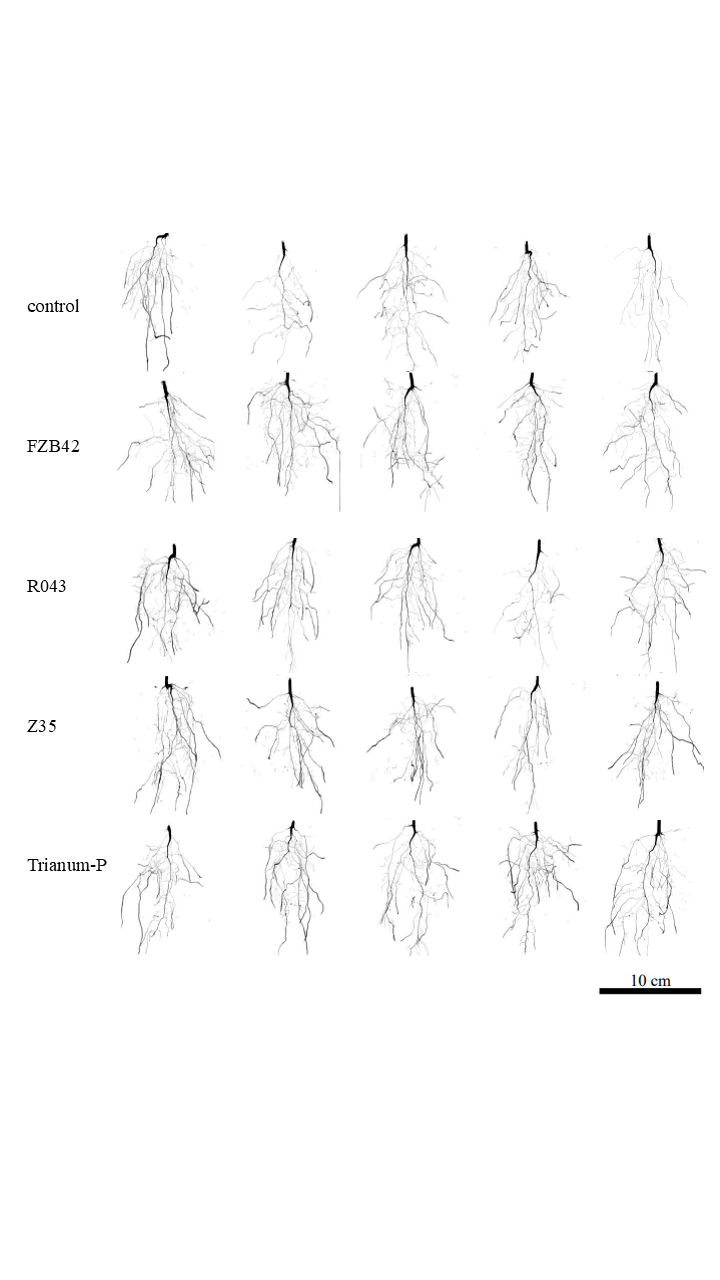

Supplement: fiaf063_Supplemental_Files [file fiaf063_supplemental_files.zip › Fig S2.tif]
